# The Characteristics and Follow-Up of SARS-CoV-2 Infection in Pediatric Oncology Patients

**DOI:** 10.7759/cureus.46149

**Published:** 2023-09-28

**Authors:** Raghad Alkharouby, Noura Aljehani, Nasser Alsubaie, Anas Alqarni, Mohammed Hariri, Naglla Elimam, Baraa AlhajHussein, Aeshah A AlAzmi

**Affiliations:** 1 College of Medicine, King Saud Bin Abdulaziz University for Health Sciences, Jeddah, SAU; 2 College of Medicine, University of Bisha, Bisha, SAU; 3 College of Medicine, University of Jeddah, Jeddah, SAU; 4 Department of Pediatric Oncology Hematology, Princess Noorah Oncology Center, King Abdulaziz Medical City, Jeddah, SAU; 5 Department of Pathology and Laboratory Medicine, King Abdulaziz Medical City, Jeddah, SAU; 6 Department of Pharmaceutical Care Services, Princess Noorah Oncology Center, King Abdulaziz Medical City, Ministry of National Guard Health Affairs, Jeddah, SAU; 7 Department of Pediatric Oncology Hematology, Bone Marrow Transplant (BMT), Princess Noorah Oncology Center, King Abdulaziz Medical City, Ministry of National Guard Health Affairs, Jeddah, SAU; 8 King Abdullah International Medical Research Center, King Saud Bin Abdulaziz University for Health Sciences, Jeddah, SAU

**Keywords:** pediatric, oncology, pediatric oncology, covid 19, sars-cov-2

## Abstract

Introduction

Clinical data about the first and second most prominent waves of SARS-CoV-2 among pediatric cancer patients were inconsistent. This study aims to retrospectively report the clinical characteristics and outcomes of SARS-CoV-2 infection in pediatric oncology patients.

Methods

This is an observational, retrospective study conducted in a tertiary care oncology center from March 2020 to May 2022. We reviewed the prevalence, severity of symptoms, and duration of positivity in relation to blood count laboratory data and mortality with a follow-up of 30 days post-infection for SARS-CoV-2.

Results

A total of 396 PCR tests were performed on 342 pediatric cancer patients. The overall rate of SARS-CoV-2 positivity was 43.1% (2.7% in the first wave and 95.4% in the second wave). Among 342 screened pediatric cancer patients, 72 patients had confirmed SARS-CoV-2 positivity in 92 different episodes. Nearly 59% had a mild or moderate infection, with fever and cough as the predominant presentations. The mean duration of positivity was 18.4±7.76 days. Comparing the laboratory values before and after acquiring the COVID-19 infection, only monocytes, hemoglobin, hematocrit, and platelets were statistically significantly affected, with P-values of 0.002, 0.03, 0.02, and 0.01, respectively. More than 18% of patients had grade 3 to 4 neutropenia (absolute neutrophil count=0.39±0.35) before COVID-19 infection and remained neutropenic throughout the disease, regardless of symptom severity. The mean recovery time was 13.67±8 days, which resulted in a delay in cancer treatment delivery of up to four weeks in 42.2% of patients.

Conclusion

Our data demonstrated that pediatric cancer patients with SARS-CoV-2 infection have a mild to moderate course of COVID-19 disease, with the majority being symptomatic, yet a great portion of our study population experienced treatment interruptions reaching up to four weeks caused by COVID-19.

## Introduction

In December 2019, the first case of severe acute respiratory syndrome coronavirus 2 (SARS-CoV-2) was confirmed in Wuhan, China. Since then, COVID-19 infections have spread rapidly and have affected all ages worldwide [1, 2]. In Saudi Arabia, COVID-19 had reached its peak by March 2020, with a total of 808,742 confirmed affected cases and a 1.1% mortality rate [3]. Thus, governments set sanctions and lockdowns to limit transmission. Ever since every medical division has been affected [4], some patients were not allowed into hospitals until a viral test was negative; others were isolated, resulting in a delay in their treatment. Previous studies in Riyadh, Saudi Arabia, estimated that 63% of pediatric cancer patients reported a delay in treatment during the pandemic; they reported the appointment cancellation was due to COVID-19 precautionary measures [5].

According to early findings, adult cancer patients have an increased risk of acquiring SARS-CoV-2 infection than the general population since their cancer and its therapy have suppressed their immune systems [6, 7]. The severity of the disease, together with underlying comorbidities and the side effects of cancer treatment, all contribute to vulnerability [8]. Moreover, evidence from China suggests that SARS-CoV2-infected cancer patients have a much greater incidence of serious adverse outcomes, such as the requirement for assisted ventilation, intensive care unit (ICU) hospitalization, and death [9]. However, reports from the initial pandemic wave indicated that it was not the case with pediatric cancer patients, the majority of whom were at minimal risk for serious COVID-19 infections [10], and only presented with asymptomatic, mild or moderate clinical SARS-CoV-2 infections despite being immunocompromised [11, 12].

On the contrary, later retrospective and prospective observational studies of all UK children concluded that, compared to the general pediatric population, children with cancer did not appear to be at a greater risk of developing a severe infection from COVID-19 [10]. Moreover, another meta-analysis found no correlation between COVID-19 and increased mortality in cancer children, as the overall survival rate was 99.4% [9]. The results of a study conducted in Saudi Arabia concluded that in general pediatrics, there are no significant variations in disease severity according to gender and immunological state, yet the same investigations were not conducted in pediatric cancer [3].

Although COVID-19 had multiple waves, the two most significant ones peaked between March 2020-March 2021 and April 2021-May 2022. Available published data comparing the two waves reported different impacts on children with cancer; however, the results were inconsistent. For example, a study from India concluded that pediatric cancer patients were more severely affected by the second wave than the first [10]. In comparison, an online survey-based study revealed that the number of pediatric oncology patients who tested positive for COVID-19 between the first and second waves of the pandemic increased by five times [11]. Therefore, this study aims to retrospectively report the experience of COVID-19 infection in pediatric cancer patients during the COVID-19 pandemic at the Princess Noorah Oncology Center at King Abdulaziz Medical City in Jeddah, Saudi Arabia.

The findings of this study were presented as a poster abstract at the ​7th Health Professions Conference held at King Saud bin Abdulaziz University for Health Sciences - Jeddah, Saudi Arabia.

## Materials and methods

This is an observational, analytical, retrospective study of all pediatric patients diagnosed with cancer at King Abdulaziz Medical City-Jeddah, Princess Noorah Oncology Center (PNOC). This study evaluated the prevalence of developing COVID-19, the severity of COVID-19 infection symptoms, and the duration of positivity in relation to blood count laboratory data (including white blood cell (WBC) count, absolute neutrophil count (ANC), lymphocyte count, monocyte count, hemoglobin, hematocrit, platelet) and mortality with a follow-up of 30 days post-infection. SARS-CoV-2 nasal/oropharyngeal swabs were tested using real-time reverse transcriptase polymerase chain reaction (RT-PCR) from March 2020 to March 2021 (the first wave) and April 2021 to May 2022 (the second wave). Data was abstracted from the computerized physician order entry (CPOE) by accessing patient demographic information, lab reports, underlying comorbidities, recovery time, and treatment location (inpatient, outpatient, emergency department). The pre-test refers to the most recent laboratory result obtained within one month prior to COVID-19 acquisition, while the post-test measures the lowest decline from the baseline that occurs before recovery.

Inclusion and exclusion criteria

Our study included pediatric cancer patients aged 0-14, who were diagnosed with cancer at the Princess Noorah Oncology Center (PNOC) and had a confirmed laboratory diagnosis of COVID-19. We excluded any cancer patients older than 14 years.

Definition and diagnosis criteria

Episodes defined as positive SARS-CoV-2 samples in one distinct hospital visit before discharge to home care. King Abdulaziz Medical City’s (KAMC) Multidisciplinary Management Guidelines for COVID-19 provide a severity score based on treatment requirements. The score categorizes individuals into four groups: asymptomatic, mild, moderate, and severe. Asymptomatic individuals show no symptoms of COVID-19 at any point. Mild cases do not require hospitalization for COVID-19 symptoms. Moderate cases require inpatient management for COVID-19 symptoms. Severe cases require intensive care unit (ICU)-level care for COVID-19 symptoms. This severity score can help healthcare professionals determine the appropriate level of care and treatment for individuals with COVID-19 [13].

Participants’ confidentiality was assured; no identifiers or any personal information were collected, and all hard and soft copies of data were kept in a secure place within Ministry of National Guard Health Affairs (MNGHA), King Abdulaziz Medical City (KAMC) premises with access by the research team only. The study protocol was approved by the Institutional Review Board (approval no. RSS22J-005-07).

## Results

During the study period, all children with cancer who presented to the hospital were routinely screened for COVID-19 infections, and a total of 342 pediatric cancer patients met the inclusion criteria (206 children presented and were screened during the first wave and 136 children presented and were screened during the second wave of the COVID-19 pandemic).

Rate of SARS-CoV-2 positivity

A total of 396 (223 in the first wave and 173 in the second wave) RT-PCR tests were performed for all 342 pediatric patients. The age of children with samples taken for SARS-CoV-2 screening during the first and second waves was younger than 6 years (141 versus 99), 6 to 12 years (67 versus 63), and older than 12 years (15 versus 11), respectively.

The overall rate of SARS-CoV-2 positivity was 43.1% in 171 out of 396 tests, with rates of 2.7% (6 out of 223 samples from 206 patients) and 95.4% (165 out of 173 samples from 136 patients) in the first and second COVID-19 waves, respectively.

After the exclusion process, 92 positive samples in different episodes (six positive samples in the first wave and 86 positive samples in the second wave) were taken from five out of 72 patients who presented and were screened in the first wave with 6.5% (six out of 92 positive samples) and 67 out of 72 patients who presented and were screened during the second wave with 93.5% (86 out of 92 positive samples) and met the inclusion criteria and were included in the analysis.

**Figure 1:**
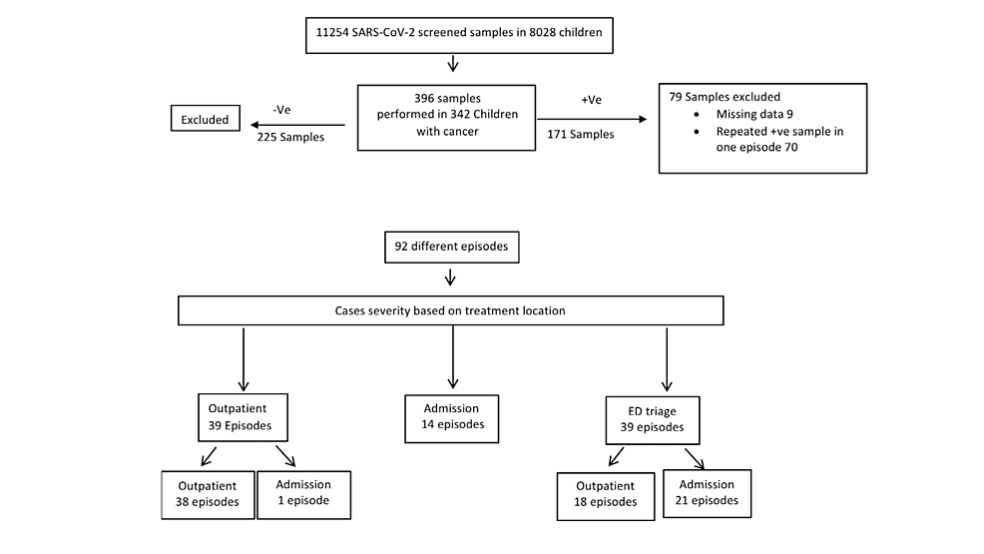
Consortium Diagram 1 *SARS-CoV-2: severe acute respiratory syndrome coronavirus 2; -Ve: Negative; +ve: Positive; ED: emergency department

All patients have follow-up data after the initial result of a SARS-CoV-2 positive test for a minimum of 30 days. The consortium diagram (Figure 1) provides details of a screened case.

Patients’ characteristics

The overall patient distribution of females to males is 1:1 ratio with a median age of 6 years (interquartile range (IQR) 4-9). Seventy-four percent of (53 out of 72) patients were receiving immunosuppressive or targeted therapy, including dinutuximab beta, ruxolitinib, cyclosporine, brentuximab, and steroids, with or without conventional chemotherapy and/or radiation therapy. Almost 21% (15 out of 72) patients received hematopoietic stem cell transplants (HSCT) within the 12 months preceding the positive SARS-CoV-2 result.

Comparing the hospitalization rate among patients presented during the first and second waves, 45% (30 out of 67 patients) of patients were hospitalized for further management, compared to 16.7% (one out of five patients) in the first wave (P-value=0.14).

Characteristic of samples with SARS-CoV-2 positivity

With regard to the type of malignancy in the confirmed SARS-CoV-2 positive samples, 66.3% (61 out of 92 samples) were from 50 patients with hematological malignancies. Leukemia was the most prevalent underlying cancer type, representing 62% (57 out of 92 samples taken from 47 patients), while positive samples from 11 patients with solid tumors represented 30.4% (28 out of 92 samples). The age of children with SARS-CoV-2 positive samples was younger than 6 years (45 out of 92 samples; 48.9%), 6 to 12 years (39 out of 92 samples; 42.4%), and older than 12 years (8 out of 92 samples; 8.7%), respectively. Table 1 provides the characteristics of included 92 samples with confirmed SARS-CoV-2 infection.

**Table 1:** Basic characteristics of confirmed cases of SARS-CoV-2 CNS: Central nervous system

Variable	N=92	%
Age at diagnosis		
≤ 6 years	47	51.09
6.1 to 12 years	38	41.30
>12 years	7	7.61
Gender		
Female	47	51.09
Male	45	48.91
Cancer type		
Malignant bone tumors	4	4.35
Soft tissue and other extraosseous sarcomas	4	4.35
Leukemias, myeloproliferative diseases, myelodysplastic diseases, and other leukemias	57	61.96
Neuroblastoma and other peripheral nervous system tumors	9	9.78
Neoplasms of histiocytes and accessory lymphoid cells	2	2.17
Renal tumors	4	4.35
CNS and miscellaneous intracranial and intraspinal neoplasms	7	7.61
Blood vessel tumors	1	1.09
Lymphomas and reticuloendothelial neoplasms	4	4.35
Treatment phase		
Naïve	4	4
Induction	8	9
Consolidation	26	28
Maintenance	34	37
Off therapy	20	22
Treatment type		
Conventional chemotherapy	57	61.96
Immunotherapy or immunosuppressant	11	11.95
Targeted therapy	3	3.26
Off treatment	17	18.48
Naïve	4	4.35

Spectrum of illness severity and outcome

Nearly 59% of screened patients had a mild or moderate type of SARS-CoV-2 acquired infection, with fever and cough as the predominant signs and symptoms at presentation, while 41% had no presenting symptoms. The overall hospitalization rate is 39% (36 out of 92 positive samples in different episodes), in which the majority of hospitalizations were recorded in children less than 6 years of age (55.6%, 20 out of 36), followed by age from 6 to less than 12 years (38.9%, 14 out of 36), while the fewest hospitalizations were recorded in adolescents (5.6%, 2 out of 36).

Among cases presented to the emergency department, 53.8% (21 out of 39) required admission for supportive care therapy and close observation, and 46.2% (18 out of 39) were discharged safely, while 42.4% (39 out of 92) cases presented in the outpatient oncology clinic for routine follow-up for their due chemotherapy cycle (2.6% were symptomatic and required admission, while 97.4% were asymptomatic and offered home isolation and discharged safely). Among hospitalized patients, 15.2% acquired COVID-19 infection during hospitalization and remained admitted for supportive care or for chemotherapy completion. Notably, the majority of SARS-CoV-2 infected patients (37%) were on maintenance, consolidation, and off-therapy treatment (37%, 28%, and 21.7%, respectively).

**Figure 2:**
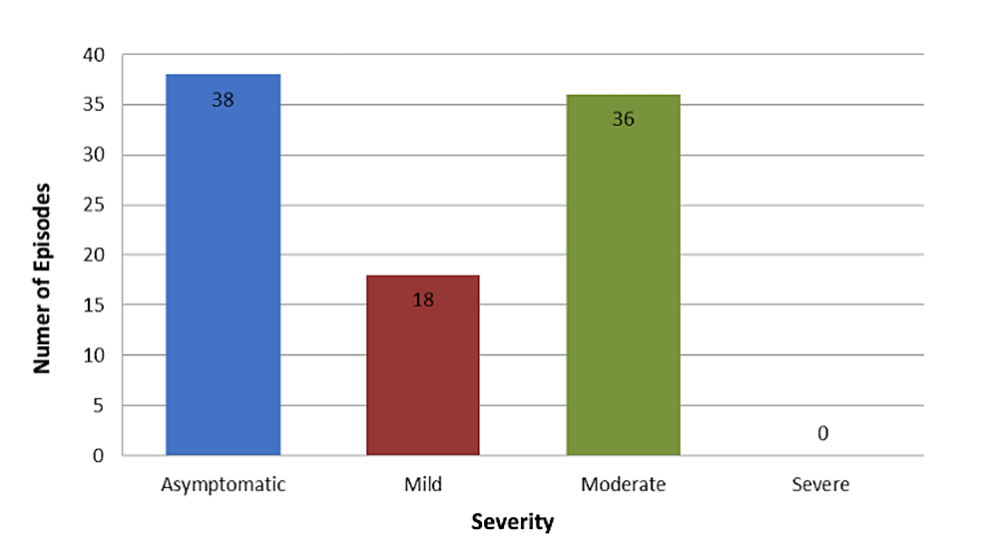
COVID-19 severity index according to treatment requirement Episodes defined as positive SARS-CoV-2 samples in one distinct hospital visit before discharge to home care.

Figure 2 and Table 2 demonstrate the absolute distribution of the COVID-19 severity index according to treatment requirements and phase.

**Table 2:** Distribution of COVID-19 cases severity according to treatment phase Episodes defined as positive SARS-CoV-2 samples in one distinct hospital visit before discharge to home care.

COVID-19 severity			
Therapy phase	Asymptomatic	Mild	Moderate
Naïve	3	0	1
Induction	2	1	5
Consolidation	6	7	13
Maintenance	15	8	11
Off therapy	12	2	6

Outcome

Examining the time to recovery, the mean duration of SARS-CoV-2 positivity was 18.4 days (SD±7.76). Noteworthy, 42% (30 out of 72) of patients experienced delay and interruption in cancer treatment delivery that ranged from one week to up to 30 days secondary to SARS-CoV-2 positivity, in which initial treatment for 11 newly diagnosed patients was delayed - in three patients for one week (two leukemia patients and one patient with Wilms tumor), and eight patients had a delay for more than one week with the following diagnoses: two patients with leukemia, two patients with neuroblastoma, one patient with osteosarcoma, one patient with neurofibromatosis type-1, one patient with an atypical teratoid rhabdoid tumor, and one patient with medulloblastoma. The remaining 19 out of 30 patients were either in the consolidation or maintenance phases and experienced delays and treatment interruptions for up to four weeks.

With regards to laboratory value findings before and after acquiring COVID-19 infection, the most prevalent laboratory finding was a decrease in the value after acquiring COVID-19 infection, with statistically significant results for monocytes (P-value=0.002), hemoglobin (P-value=0.03), hematocrit (P-value=0.02), and platelets (P-value=0.01).

Nearly 18.5% of patients with a SARS-CoV-2 confirmed test had grade three to four neutropenia before acquiring COVID-19, and they remained neutropenic throughout the COVID-19 infection with a mean ANC level of 0.39±0.35 (range 0 to 0.91). The mean time to neutrophil recovery after COVID-19 infection was 13.67±8.08 days. D-dimer was performed in one patient only, and it was mildly elevated at 2.3 (reference range: 0.17 to 0.5 mg/L).

Notably, during the study period, there was no case of fatality or need for intensive care management as a result of the COVID-19 infection. Table 3 shows laboratory data of included patients.

**Table 3:** Laboratory data of included patients

Lab Variable	Mean±SD Pre-COVID-19	Mean±SD Post-COVID-19	P-Value
White blood cell (WBC) count ×10^9^/L	7.43±16.57	5.09±15.86	1.99
Absolute neutrophil count (ANC) ×10^9^/L	4.08±7.31	1.96 ±5.86	3.57
Lymphocyte count ×10^9^/L	1.46±1.29	1.55±1.37	0.49
Monocyte count ×10^9^/L	0.80±1.17	0.47±0.75	0.002
Hemoglobin g/dL	10.64±1.95	9.63±1.63	0.03
Hematocrit %	32.32±5.34	28.99±4.73	0.02
Platelet ×10^9^/L	219.98±124.55	187.57±115.54	0.01

## Discussion

Pediatric oncology patients are predicted to be in an immunocompromised state and at higher risk for a severe COVID-19 infection, with an expectation of increases in healthcare utilization. Contradictory data are available from different countries, such as the United Kingdom, France, Spain, and the United States, describing the COVID-19 disease course in children with cancer, which ranged from mild disease to a higher risk of a severe form of COVID-19 infection with some case fatalities reported [8-18].

Our study showed that the majority of cases are mild to moderate, but the mean duration of SARS-CoV-2 positivity was 18.4 days (SD±7.76), which resulted in delaying and interrupting cancer treatment for up to 30 days.

More than half of the patients who came to the ER and had a positive COVID-19 test were admitted for further observation, and most of them were discharged safely. No intensive care was required for any of our cases, and no fatalities due to the infection were reported. This is similar to the findings from various studies, where they reported a mild course of the disease without any fatalities [8-12]. The highest hospitalization rates secondary to COVID-19 infection were reported in children aged less than five years and adolescents, while the lowest hospitalization rates were reported in children aged 5 to 11 years [14]. These findings are almost comparable to our results, where children less than six years of age required hospitalizations associated with COVID-19 compared to other screened age groups.

Hematological malignancies were the most common underlying diagnosis in most of the patients with a leukemia predominance, who, in fact, had longer treatment durations and were expected to present to the hospital more often. Data from a study in the UK showed that leukemia patients constitute a large portion of positive COVID-19 hemato-oncology pediatrics [8]. Data from Saudi Arabia reported that the majority of patients had hematological malignancies, with 60.4% being leukemia or lymphoma patients [3]. This is similar to our patient population, where hematological disease is the predominant underlying disease. A study from India concluded that pediatric cancer patients were more severely affected by the second wave than the first [10]. Another study revealed that the number of pediatric oncology patients who tested positive for COVID-19 between the first and second waves of the pandemic increased by five times [11], similar to our population, where the majority of confirmed cases with COVID-19 infection were presented in the second wave. A couple of studies showed that young children often acquire COVID-19 infection through community sources [19-21]. During the COVID-19 pandemic, the government of the Kingdom of Saudi Arabia made a critical effort and enforced very strict policies to reduce the spread of COVID-19 infection in the country. In addition to social distancing and mask-wearing, a countrywide lockdown was started on March 25, 2020, and gradually reopened by June 2020. These measures are of special importance, as the present study showed that the rate of confirmed COVID-19 cases with SARS-CoV-2 positivity increased from 2.7% in the first wave to 95.4% in the second wave. This may explain the difference in the number of confirmed cases between the two waves. However, our study findings showed that there was no association between the first and second waves of the pandemic in terms of infection severity in children with cancer.

The pandemic has significantly changed the continuation of care for children with cancer worldwide by creating barriers such as COVID-19 case prioritization in addition to lockdown and restricted transportation, which have contributed to delayed childhood cancer diagnoses and treatment [4, 5]. A great portion of our population experienced treatment interruption in different forms, reaching up to four weeks, similar to a study that has been done in Riyadh, Saudi Arabia, where 60.5% of the patients reported a delay in treatment received due to the lockdown and cancellation of their appointments [3]. A study has shown that treatment interruption has been reported in 29-44% of centers in the Middle East, North Africa, and West Asia regions [4-6]. Rouger-Gaudichon et al. found that patients with COVID-19 had profound neutropenia and lymphopenia. In our study we noticed similar findings, although it was not statistically significant as neutropenia and lymphopenia could be attributed to the type of cancer treatment that the patients are receiving, these findings were observed in the cancer patients population even without the COVID-19 infection [16]. However, monocytes, hemoglobin, hematocrit, and platelets were significantly affected in our patients after the infection compared to their values before. These results may be reassuring that children with cancer have a milder course of COVID-19 infection with better outcomes, and they might safely continue their cancer treatment.

Data showed that most children hospitalized for COVID-19 infection were not eligible for COVID-19 vaccination because of the age limit or because it was not available [21-23]. This is similar to our findings, as all children with cancer who acquired SARS-CoV-2 were hospitalized before vaccinations against COVID-19 were available to the pediatric population. Vaccinations against COVID-19 are widely available in the Kingdom of Saudi Arabia, but they were first approved for use in children on June 25, 2021, for those aged 12 and older, and then on December 21, 2021, for those aged 5 to 11 years [24-26].

Study limitations

The main limitations of this study concern the lack of national data on COVID-19 in children with cancer incidence and outcome. In addition to this, our findings represent a single-institution study with a small number of patients. Despite continuing SARS-CoV-2 positivity in our patient population of children with cancer, no severe cases or fatalities were associated with the COVID-19 infection. Our data highlight the importance of considering changes in practice with an optimal strategy to prevent delaying and interrupting cancer treatment therapy in mild-to-moderate cases, which needs to be systematically studied. Larger, multi-organizational, and nationwide studies should be conducted to validate our findings by examining the national prevalence of COVID-19 infection, symptom severity, and long-term outcome.

## Conclusions

According to the study, 43.1% of children with cancer tested positive for SARS-CoV-2, with a higher rate during the pandemic's second wave. Hematological malignancy, particularly leukemia, was the most common diagnosis. Hospitalization rates were higher among children under 6 years old who contracted COVID-19.

The study found that most children with cancer and SARS-CoV-2 had a mild to moderate COVID-19 course, with many experiencing symptoms. However, a significant number of children in the study faced treatment interruptions due to COVID-19 infection, which lasted up to four weeks. While the results suggest that children with cancer can continue their treatment safely, further research is needed to determine the long-term effects of treatment delays and interruptions.
